# Myocardial injury and fibrogenesis: extracellular volume expansion is associated with elevated Galectin-3 levels in patients with myocarditis

**DOI:** 10.1186/1532-429X-16-S1-P290

**Published:** 2014-01-16

**Authors:** Lukas Radziwolek, Ulf K Radunski, Katharina Koopmann, Sebastian Bohnen, Tanja Zeller, Gunnar Lund, Aljoscha D Krull, Nina Hauschild, Christian Stehning, Gerhard Adam, Stefan Blankenberg, Kai Muellerleile

**Affiliations:** 1 Cardiology, University Heart Center, Hamburg, Germany; 2 Radiology, University Medical Center, Hamburg, Germany; 3 Philips Research, Hamburg, Germany

## Background

Myocarditis subsumes a variety of entities, including diverse courses from complete healing to dilated cardiomyopathy with severe myocardial fibrosis. T1-mapping cardiovascular magnetic resonance (CMR) has the ability to quantify myocardial extracellular volume (ECV) as a surrogate of acute and chronic myocardial injury. Galectin-3 is an important mediator of fibrogenesis and contributes to adverse left ventricular (LV) remodeling. This study evaluated, if myocardial ECV expansion is linked to Galectin-3 levels in patients with myocarditis.

## Methods

Galectin-3 blood levels were measured in 20 patients with myocarditis using a commercially available chemiluminescent microparticle immunoassay (ARCHITECT Galectin-3, Abbott Germany). T1 quantification was performed at 1.5 Tesla using the modified Look-Locker inversion-recovery (MOLLI) sequence before and 15 minutes after administration of 0.075 mmol/kg gadolinium-BOPTA. Global myocardial ECV was calculated from T1 maps generated by a dedicated plug-in written for the OsiriX software.

## Results

Median Galectin-3 level was 17.4 ng/mL (interquartile range 13.2 to 20.5 ng/mL) and median global myocardial ECV was 29 % (interquartile range 26 to 33 %) in the study population. There was a significant correlation between Galectin-3 levels and global myocardial ECV (r = 0.50; p < 0.05). In contrast, no significant correlation was found between Galectin-3 levels and LV end-diastolic volumes (r = -0.08; p = ns), LV end-systolic volumes (r = 0.06; p = ns), LV stroke volumes (r = -0.33; p = ns); LV ejection fractions (r = -0.11; p = ns), Troponin T levels (r = 0.20; p = ns) or NT-proBNP levels (r = 0.28; p = ns), respectively.

## Conclusions

Myokardial ECV expansion, as a surrogate for myocardial injury, is associated with increased Galectin-3 levels, indicating activated fibrogenesis in patients with myocarditis. Combining Galectin-3 measurements with ECV-imaging could improve risk stratification beyond conventional imaging parameters or biomarkers in these patients.

## Funding

Marija-Orlovic-Foundation.

